# Rutin and Hesperidin Revoke the Hepatotoxicity Induced by Paclitaxel in Male Wistar Rats *via* Their Antioxidant, Anti-Inflammatory, and Antiapoptotic Activities

**DOI:** 10.1155/2023/2738351

**Published:** 2023-05-26

**Authors:** Yasmine A. Ali, Hanan A. Soliman, Mohamed Abdel-Gabbar, Noha A. Ahmed, Kandil A. A. Attia, Fatma M. Shalaby, El-Shaymaa El-Nahass, Osama M. Ahmed

**Affiliations:** ^1^Biochemistry Department, Faculty of Science, Beni-Suef University, P.O. Box 62521, Beni-Suef, Egypt; ^2^Physiology Division, Zoology Department, Faculty of Science, Beni-Suef University, P.O. Box 62521, Beni-Suef, Egypt; ^3^Clinical Nutrition Department, College of Applied Medical Sciences, Jazan University, P.O. Box 114, Jazan 45142, Saudi Arabia; ^4^Department of Evaluation of Natural Resources, Environmental Studies and Research Institute, El-Sadat City University, El-Sadat City 32897, Egypt; ^5^Biology Department, Faculty of Science, King Khalid University, Abha, Saudi Arabia; ^6^Department of Zoology, Faculty of Science, Mansoura University, Mansoura, Egypt; ^7^Department of Pathology, Faculty of Veterinary Medicine, Beni-Suef University, P.O. Box 62521, Beni-Suef, Egypt

## Abstract

Paclitaxel, one of the most effective chemotherapeutic drugs, is used to treat various cancers but it is exceedingly toxic when used long-term and can harm the liver. This study aimed to see if rutin, hesperidin, and their combination could protect male Wistar rats against paclitaxel (Taxol)-induced hepatotoxicity. Adult male Wistar rats were subdivided into 5 groups (each of six rats). The normal group was orally given the equivalent volume of vehicles for 6 weeks. The paclitaxel-administered control group received intraperitoneal injection of paclitaxel at a dose of 2 mg/Kg body weight twice a week for 6 weeks. Treated paclitaxel-administered groups were given paclitaxel similar to the paclitaxel-administered control group together with oral supplementation of rutin, hesperidin, and their combination at a dose of 10 mg/Kg body weight every other day for 6 weeks. The treatment of paclitaxel-administered rats with rutin and hesperidin significantly reduced paclitaxel-induced increases in serum alanine transaminase, aspartate transaminase, lactate dehydrogenase, alkaline phosphatase, and gamma-glutamyl transferase activities as well as total bilirubin level and liver lipid peroxidation. However, the levels of serum albumin, liver glutathione content, and the activities of liver superoxide dismutase and glutathione peroxidase increased. Furthermore, paclitaxel-induced harmful hepatic histological changes (central vein and portal area blood vessel congestion, fatty changes, and moderate necrotic changes with focal nuclear pyknosis, focal mononuclear infiltration, and Kupffer cell proliferation) were remarkably enhanced by rutin and hesperidin treatments. Moreover, the elevated hepatic proapoptotic mediator (caspase-3) and pro-inflammatory cytokine (tumor necrosis factor-*α*) expressions were decreased by the three treatments in paclitaxel-administered rats. The cotreatment with rutin and hesperidin was the most effective in restoring the majority of liver function and histological integrity. Therefore, rutin, hesperidin, and their combination may exert hepatic protective effects in paclitaxel-administered rats by improving antioxidant defenses and inhibiting inflammation and apoptosis.

## 1. Introduction

Paclitaxel, which stabilizes microtubules and inhibits their depolymerization during cell division, is one of the most widely used chemotherapy drugs [1–4]. The active compound selection program founded by the National Cancer Institute in 1981 proved that paclitaxel was the only active biological ingredient that falls within this category and meets the standard that could be effectively used to manage cancer, mainly from clinical trials [5, 6]. Paclitaxel is used to treat various cancers, including breast, prostate, bladder, cervical, and brain cancer [7–10]. Many different cancers are also treated with paclitaxel, such as aggressive and metastatic breast cancer, ovarian cancer, lung cancer, pancreatic cancer, and others [11]. However, its administration causes numerous adverse effects, including neuropathy, cardiotoxicity, and hepatotoxicity, as well cancer cells' resistance to paclitaxel chemotherapy [12–14]. Paclitaxel has been widely known to stimulate apoptosis. Moreover, it has been recognized to produce reactive oxygen species (ROS) that trigger mitochondrial dysfunction to release cytochrome C into the cytoplasm and activate the caspase cascade and apoptosis stimulation [15, 16]. Paclitaxel promotes oxidative stress, decreases antioxidants, increases liver enzymes, and impairs renal function, which may be due to its mechanism of action and the oxidative stress that it caused [17]. Paclitaxel exacerbates liver damage during treatment and causes severe liver necrosis that may lead to mortality [18–20]. Paclitaxel has been reported to exert inflammatory actions. It also revealed a significant increase in pro-inflammatory cytokines, such as interleukin (IL)-17A, tumor necrosis factor-alpha (TNF-*α*), interferon-*γ* (IFN-*γ*), and keratinocyte, in paclitaxel-treated mice [21].

To reduce the toxicity of various organs from chemotherapeutic drugs, several studies have investigated the use of natural compounds that have antioxidant and antiapoptotic effects [22–28]. Citrus species are considered to be among the most economically significant biological resources, as they contain a variety of plant nutrients and phytochemicals with promising therapeutic properties [29]. Flavonoids have various biological effects and may confer health benefits *via* different mechanisms through anti-inflammatory, antioxidant, antimicrobial, and antiproliferative regulatory activities [30–32]. Several natural antioxidants have been experimentally tested for their potential to protect the liver, such as rutin [33] and hesperidin [34]. Combining rutin with other drugs can reduce drug resistance and side effects of chemotherapy [35]. Rutin has tremendous medicinal potential to regulate several cell signaling and apoptotic pathways implicated in cancer progression [36]. Additionally, it induces an important mechanism in inhibiting cell proliferation in neoplastic cells in the liver tissue by hepatocellular marker enzyme and tumor incursion suppression [37]. Rutin has shown remarkable protection against acrylamide-induced oxidative deoxyribonucleic acid (DNA) damage, which may be due to its antioxidant potential [38]. Hesperidin possesses chemopreventive potential against paclitaxel-induced hepatotoxicity probably by reducing oxidative stress, inflammation, apoptosis, and autophagy [39]. Furthermore, the pretreatment of hesperidin offers powerful protective effects against cisplatin-induced hepatic damage, which is achieved by its antioxidant, anti-inflammatory, and antiapoptotic activities [40]. Hesperidin's anticancer potential is controlled by ROS-dependent apoptotic pathways in certain cancer cells, despite the fact that it can be an excellent ROS scavenger and could operate as a powerful antioxidant defense mechanism [41].

Chemotherapeutic drugs such as paclitaxel have several deleterious side effects including liver injury and we aim to minimize these effects by using plant constituents with antioxidant and anti-inflammatory activities. Therefore, this research aimed to scrutinize the preventative efficacy of rutin, hesperidin, and their combination on paclitaxel (Taxol)-induced liver toxicity, as well as to investigate the roles of inflammation, oxidative stress, and apoptosis modulations in preventive action.

## 2. Materials and Methods

### 2.1. Chemicals

The trade name drug, paclitaxel, or Taxol, in the formulation vehicle of cremophor® EL*∗*(CrEL) (polyoxyethylated castor oil) (batch number: 7E05628), was obtained from Bristol-Myers Squibb global biopharmaceutical company (Princeton, USA). Rutin (batch number: 501) was obtained from Oxford Laboratory Company (Mumbai, India). Rutin is a light yellow crystalline powder with the empirical formula C27H30O16 and a molecular weight of 610.5 and tastes slightly bitter. It has low solubility in water (125 mg/L), while it is highly soluble in polar solvents and melts at around 176–178°C. Hesperidin (lot number: # SLBT3541) was obtained from Sigma-Aldrich Company (St. Louis, MO, USA). Hesperidin is a light yellow crystalline powder with the empirical formula C_28_H_34_O_15_ and a molecular weight of 610.6, odorless, and tasteless. It demonstrated poor, pH-independent, aqueous solubility, while it dissolves in dimethyl formamide and formamide at 60°C and slightly soluble in other polar solvents and melts at around 258–262°C. Alanine transaminase (ALT) reagent kit (catalog number: M11533c-21) and aspartate transaminase (AST) reagent kit (catalog number: M11531c-21) were purchased from Biosystem S.A. (Barcelona, Spain). The alkaline phosphatase (ALP) reagent kit and gamma-glutamyl transferase (GGT) reagent kit were purchased from Biosystem S.A. (Barcelona, Spain), with catalog numbers M11592-0610 and M11584c-11, respectively. A lactate dehydrogenase (LDH) reagent kit (catalog number: MX41214) was purchased from Spin React (Girona, Spain). Total bilirubin reagent kit (catalog number: 10742) and albumin reagent kit (catalog number: 10560) were purchased from HUMAN Gesellschaft für Biochemica und Diagnostica mbH (Wiesbaden, Germany). Chemicals of oxidative stress including trichloroacetic acid (TCA) (batch number: 5O011689) obtained from PanReac AppliChem ITW Companies (Spain); thiobarbituric acid (TBA) (batch number: L 16A/1916/1212/13) was obtained from Sd Fine Chem Limited (SDFCL) Company (India); 1,1,3,3 tetra-methoxy propane or malondialdehyde (MDA) (catalog number: T9889) was obtained from Sigma-Aldrich (MO, USA); metaphosphoric acid (batch number: M21519) was obtained from ALPHA CHEMIKA Company (India); 5,5- dithiobis nitrobenzoic acid (DTNB or Ellman's reagent) (batch number: 40K3652) was obtained from Sigma-Aldrich (MO, USA); Reduced glutathione (GSH) (batch number: 3W010085) was obtained from PanReac AppliChem ITW Companies (Spain); and pyrogallol (batch number: 1280B251114) was obtained from ResearchLab Company (India).

### 2.2. Experimental Animals

The experimental animals in this study were thirty adult male Wistar rats weighing 130–150 g and aged 7–8 weeks. They came from the National Research Center's Animal House in Dokki, Giza, Egypt. The animals were monitored for 15 days before the trial began to ensure that no inter competitive infections existed. The animals were kept in polypropylene cages with well-ventilated stainless steel lids at room temperature (25 ± 5°C) and on a 12-hourlight-dark cycle every day. The animals had unlimited access to water and were fed a well-balanced meal *ad libitum* daily. The Experimental Animal Ethics Committee's rules and guidelines were followed in all animal procedures. Faculty of Science, University of Beni-Suef, Egypt (Ethical Approval Number: BSU/FS/2017/8). Every effort has been made to reduce pain, distress, and discomfort among animals.

### 2.3. Experimental Design

Adult male Wistar rats were subdivided into 5 groups in this study (6 rats per group).

- Normal group: rats in this group were orally administered with 5 mL 1% carboxymethylcellulose (CMC) (vehicle in which rutin and hesperidin are dissolved)/Kg body weight (b. wt) every other day and 2 mL isotonic saline (0.9% NaCl) (vehicle in which paclitaxel is dissolved)/Kg *b*. wt twice per week *via* the intraperitoneal (i.p.) route for 6 weeks.
- Paclitaxel-administered control group: this group of rats received paclitaxel at a dose of 2 mg/Kg *b*. wt (in 2 mL 0.9% NaCl) by i.p. injection [42] twice a week on the 2^nd^ and 5^th^ days of each week for 6 weeks, an equivalent dose of 1% CMC (5 mL/Kg *b*. wt) was also given orally every other day.
- Paclitaxel-administered group treated with rutin: this group of rats received paclitaxel as in the paclitaxel-administered control group, as well as rutin orally every other day at a dose of 10 mg/Kg *b*. wt [43] (dissolved in 5 mL of 1% CMC) for 6 weeks.
- Paclitaxel-administered group treated with hesperidin: this group of rats received paclitaxel as in the paclitaxel-administered control group, as well as hesperidin orally every other day at a dose of 10 mg/Kg *b*. wt [44] (dissolved in 5 mL of 1% CMC) for 6 weeks.
- Paclitaxel-administered group treated with rutin and hesperidin combination: this group of rats received paclitaxel as in the paclitaxel-administered control group, as well as rutin and hesperidin combination orally every other day at a dose of 10 mg/Kg *b*. wt (dissolved in 5 mL of 1% CMC) for 6 weeks.

### 2.4. Blood and Liver Sampling

Under inhalation anesthesia [45], blood samples were collected from the jugular vein into gel and clot activator tubes after a 6-week treatment with the prescribed dosages. Blood samples were allowed to clot at room temperature and then centrifuged for 15 minutes at 3,000 rounds per minute (rpm). For various biochemical experiments, sera were quickly separated, split into four portions for each animal, and kept at −30°C. Following decapitation and dissection, livers were dissected for biochemical testing and histopathological examination, with each rat's liver tissue being quickly weighed and washed with isotonic saline (0.9% NaCl). A part of the liver was preserved in buffered formalin for 24 hours, then cut and placed in 70% alcohol for histopathologic analysis. The Teflon homogenizer (Glas-Col, Terre Haute, IND, USA) was used to homogenize approximately 0.5 g of each liver tissue into 5 mL 0.9% NaCl. The homogenates were then centrifuged for 15 minutes at 3,000 rpm, and the supernatants were aspirated and frozen at −30°C until employed in the assessment of oxidative stress marker-related biochemical and antioxidant parameters.

### 2.5. Determination of Liver Function Biomarkers in Serum

ALT and AST activities were assessed according to the method of Gella et al. [46]. The activities of GGT and ALP were assayed using the methods of Schumann et al. [47] and Schumann et al. [48], respectively. The activity of LDH was measured as previously described by Pesce [49]. The levels of serum albumin and total bilirubin were measured according to the procedures of Doumas et al. [50] and Jendrassik [51], respectively.

### 2.6. Liver Oxidative Stress and Antioxidant Biomarkers' Analysis

Chemical reagents prepared in the laboratory were used to evaluate liver oxidative stress and antioxidant biomarkers. The method provided by Preuss et al. [52] was used to estimate liver lipid peroxidation (LPO). Briefly, 0.15 mL 76% TCA was added to 1 mL liver homogenate to precipitate the protein. The isolated supernatant was then color-enhanced with 0.35 mL TBA. At 532 nm, the produced pale pink color was identified after 30 minutes in an 80°C water bath. The standard was MDA. On the other hand, GSH concentration in the liver was evaluated by adding 0.5 mL DTNB or Ellman's reagent (as a color-developing agent), and phosphate buffer solution (pH, 7) to homogenate supernatant after protein precipitation by centrifugation, as described by Beutler et al. [53]. At 412 nm, the generated yellow colors in the samples and GSH standard were measured and compared to a blank. The activity of liver GPx was determined using a modified version of the procedure described by Matkovics et al. [54]. The remaining GSH after it has been converted by the enzyme to GSSG (oxidized glutathione) and deducting the residual from the total is the basis of this approach. Briefly, 50 *μ*L of homogenate supernatant was introduced to a Wasserman tube that already contained 350 *μ*L of Tris buffer (pH 7.6), 50 *μ*L of GSH solution (2 mM), and 50 *μ*L of hydrogen peroxide (H_2_O_2_) (3.38 mM). The previously mentioned technique for determining GSH was used to quantify the residual GSH content at 430 nm following a 10-minute incubation period. The standard test was made using 50 *μ*L of dist. H_2_O instead of 50 *μ*L sample and the blank test was made with 100 *μ*L of distilled water instead of 50 *μ*L sample and 50 *μ*L GSH solution. Following the discovery of residual GSH in the sample, the enzyme activity was measured by converting GSH to GSSG. The activity of the liver SOD was measured using the method of Marklund and Marklund [55]. SOD inhibits pyrogallol autoxidation, which is the basis for the reaction. Superoxide ions are necessary for the process to take place. One unit of enzyme is equivalent to the quantity of enzyme required to reduce extinction changes by 50% in one minute as compared to the control.

### 2.7. Histological Investigations

After the fast decapitation and dissection of each rat, 3 mm^3^ pieces of liver from all groups were preserved in 10% neutral phosphate-buffered formalin (pH 7.2) for 24 hours. The fixed livers were transferred to the Pathology Department of Beni-Suef University's Faculty of Veterinary Medicine in Egypt for additional processing, wax blocking, sectioning, and hematoxylin and eosin (H&E) staining [56]. Histological scores were determined by examining the stained liver sections. Six random fields were estimated for each section. The number of sections in each group is six. Degenerative change, fatty change, inflammatory cell infiltration, necrosis, vascular congestion, and Kupffer cell proliferation were among the graded lesions. Scoring of these hepatic lesions was calculated based on Khafaga et al. [57] and Wasef et al. [58] and graded as follows 0 = none; 1 ≤ 25%; 2 = 26–50%; 3 = 51–75%; and 4 = 76–100%.

### 2.8. Immunohistochemical Investigations of Caspase-3 and TNF-*α*

The liver samples, secured with 10% neutral buffered formalin, were processed, blocked, and divided into 5-*μ*m–thick sections that were fixed on positive-loaded slides (Fisher Scientific, Pittsburgh, PA, USA) at the National Cancer Institute's Pathology Department. The immunohistochemical reactions in the liver sections were investigated according to the method described by previous publications [59–63]. Briefly, after antigen retrieval, liver sections were incubated for 1 hour with diluted primary antibodies (dilution: 1–100 in phosphate buffer saline) for caspase-3 or TNF-*α* (Santa Cruz Biotechnology, Santa Cruz, CA, USA). Diluted biotinylated secondary antibodies (dilution: 1–200 in phosphate buffer saline) of DakoCytomation Kit were added and incubation was carried out for 15 minutes at 37°C. Then, using a DakoCytomation Kit, horseradish peroxidase conjugated with streptavidin was added and incubated for another 15 minutes. A reaction of 3,3′-diaminobenzidine (DAB) substrate was used to visualize the bound antibody complex, which was counterstained with hematoxylin. Immunostaining was comparable across all research groups since all liver slices were incubated under the same conditions with the same antibody dilutions and for the same period. A light microscope was used to examine the immunostained liver sections and determine the degree of cell immunopositivity. A digital camera was used to capture photos of the liver section (Leica, DM2500M Leica, Wetzlar, Germany). ImageJ (1.51d), a free software program, was used to measure the area percentage of immune positivity for caspase-3 and TNF-*α* reactions according to Khafaga et al. [64] and El-Far et al. [65].

### 2.9. Statistical Analysis

The mean and standard error of the mean (SEM) were used to express all of the data. The Statistical Package for Social Sciences computer software (SPSS) (version 22, IBM software, Armonk, NY, USA) was used to perform the statistical analysis. A one-way analysis of variance (ANOVA) test was performed to clarify the significance among group means, followed by Tukey's post hoc test to compare-averaged aged results. At *p* < 0.05, differences were considered significant. Percentage changes were calculated using the formula: % change = [(Final value – Initial value)/Initial] × 100 [66].

## 3. Results

### 3.1. Effects on Serum Parameters Related to Liver Function

The serum AST, ALT, GGT, LDH, and ALP activities, as well as the total bilirubin level, increased significantly (*p* < 0.05) after rats were given paclitaxel intraperitoneally for 6 weeks. When compared to the corresponding normal controls, paclitaxel administration resulted in a significant decrease in serum albumin level, with a documented percentage change of −37.37%. The treatment of paclitaxel-administered rats with rutin and/or hesperidin resulted in substantial decreases in increased serum AST, ALT, LDH, ALP, GGT, and total bilirubin levels when compared to the paclitaxel-administered control group. The treatment with rutin and its combination with hesperidin, on the other hand, resulted in a significant change in albumin levels, with recorded percentage changes of +31.72 and + 34.41%, respectively, whereas the treatment with hesperidin produced a nonsignificant improvement (*p* > 0.05). Moreover, compared with the paclitaxel-administered control group, the treatment of paclitaxel-administered rats with rutin and hesperidin combination was the most efficacious in improving the elevated serum AST, ALT, LDH, ALP, and total bilirubin levels, as well as the decreased albumin levels. Hesperidin treatment was the most effective in lowering GGT activity, with a recorded percentage change of −33.33% (Table 1).

### 3.2. Effects on Liver Oxidative Stress and Antioxidant Defense Parameters

Paclitaxel was given intraperitoneally to rats for six weeks, resulting in a highly significant rise in liver LPO and a highly significant decrease in liver GSH content, as well as SOD and GPx activities. The treatment of paclitaxel-administered rats with rutin, hesperidin, and their combination significantly decreased liver LPO. Hesperidin seemed to be the most effective in lowering the increased LPO product in the liver. In contrast to the paclitaxel-administered control group, paclitaxel-administered rats treated with rutin, hesperidin, or their combination showed a significant improvement in lowered liver SOD and GPx activities. The treatment of paclitaxel-administered rats with rutin and hesperidin caused a significant increase in the GSH content (Table 2).

### 3.3. Liver Histological Changes

**Figure 1:**
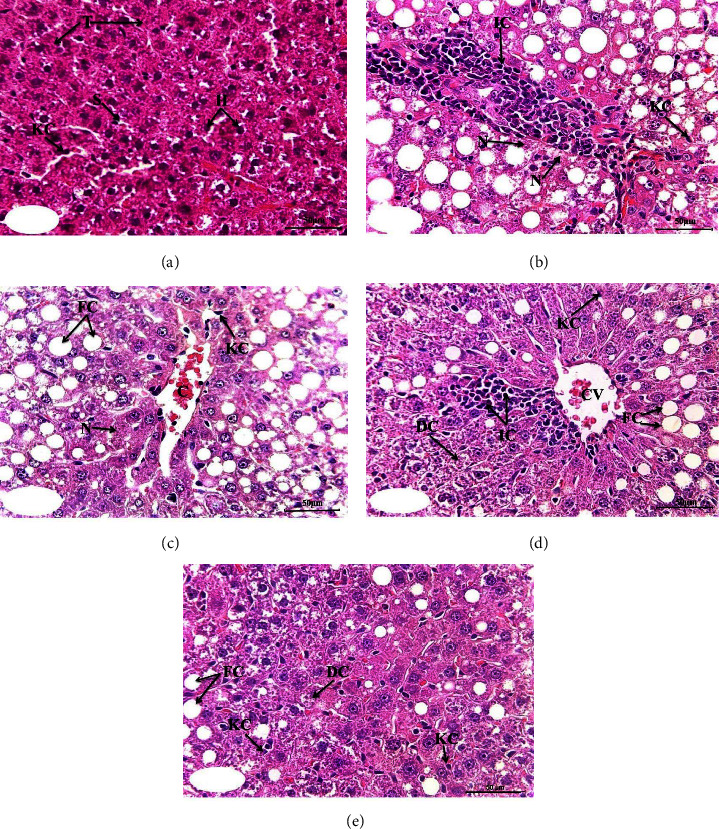
Photomicrographs of liver sections of the normal (a), paclitaxel-injected control group (b), and paclitaxel-injected groups treated with rutin (c), hesperidin (d), and their combination (e). (H) hepatocytes; (T) trabeculae; (S) sinusoids; and KC: Kupffer cells; (N) necrosis; IC: inflammatory cells infiltration; FC: fatty changes; (C) congestion; CV: central vein; DC: degenerative changes. **(**H&E; ×400).

Histopathological findings of the liver specimens from different experimental groups are presented in Figure 1 and Table 3. The normal group's liver sections revealed normal histological structures in the form of a thin-walled central vein and normal hepatocytes forming the hepatic cords radiating from the central vein toward the periphery and alternating with narrow blood spaces, the sinusoids, which are lined with single-layered Kupffer cells on histopathological analysis (Figure 1(a)). Conversely, the livers of the paclitaxel-administered group showed marked pathological changes in the form of central vein and portal area blood vessel congestion, marked degenerative changes, including fatty changes and moderate necrotic changes with focal nuclear pyknosis in certain areas, focal leukocytic infiltration (mainly mononuclear cells), and Kupffer cell proliferation (Figure 1(b)). These changes were altered to some extent in different paclitaxel-treated groups. These changes were amended to some extent by treatments of paclitaxel-administered groups. First, rats treated with paclitaxel/rutin showed severe degenerative and fatty changes associated with moderate necrotic changes and focal leukocytic infiltration associated with moderate proliferation of Kupffer cell activation (Figure 1(c)). Second, pathologic changes in the paclitaxel/hesperidin-treated group were relatively similar to those in the paclitaxel/rutin-treated group (Figure 1(d)). Finally, the treatment of paclitaxel/rutin/hesperidin produced a good improvement in liver histological changes compared with other treated rats. Moderate degenerative changes and mild necrotic changes accompanied by the mild Kupffer cell proliferation were noted (Figure 1(e)). The significantly elevated histological lesion scores of degenerative changes, fatty changes, necrosis, inflammatory cells, congestion, and activated Kupffer cell proliferation in the paclitaxel-injected group were significantly decreased by treatments with rutin, hesperidin, and their combination. The combinatory treatment was the most effective in improving the degenerative and fatty changes (Table 3).

### 3.4. Effects on Liver Caspase-3 and TNF-*α*

**Figure 2:**
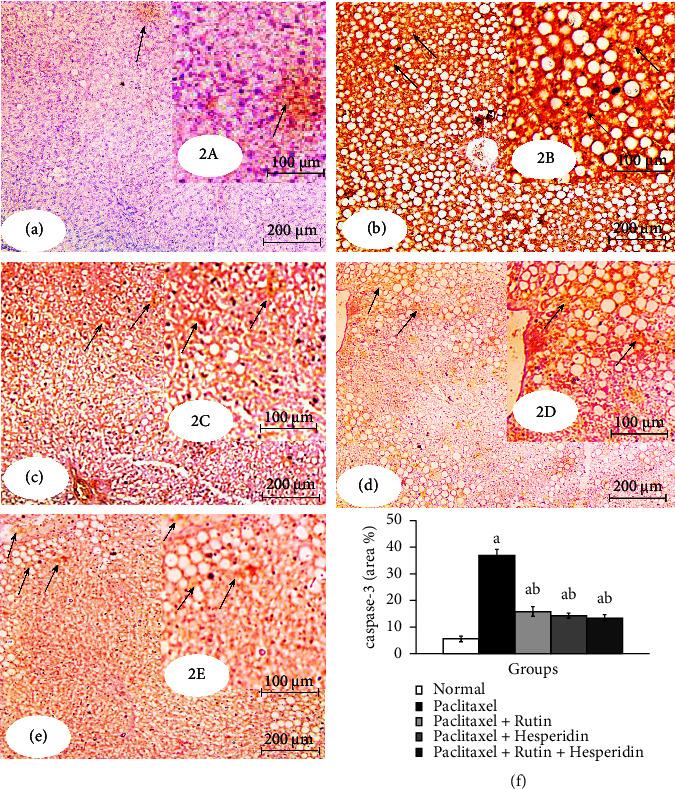
Photomicrographs of immunohistochemically stained liver sections for caspase-3 detection showing very weak expression in normal (2a and 2A), very strong expression in the paclitaxel-administered group (2b and 2B), and moderate expression in paclitaxel-administered groups treated with rutin (2c and 2C), hesperidin (2d and 2D), and their combination (2e and 2E). Arrows indicate positive reactivity. 2f indicates the image analysis result of caspase-3 of the tested groups. ^*a*^*p* < 0.05: significant compared with the normal group. ^*b*^*p* < 0.05: significant compared with the paclitaxel-injected group. Photomicrographs 2A–2E are magnified sectors of Photomicrographs 2a–2e respectively.

**Figure 3:**
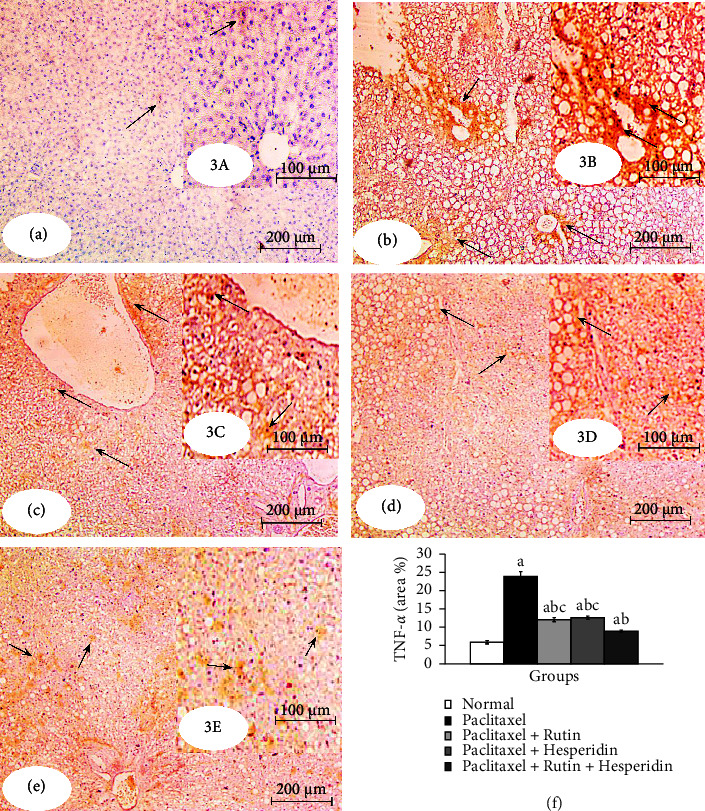
Photomicrographs of immunohistochemically stained liver sections for TNF-*α* detection showing very weak expression in normal (3a and 3A), strong expression in the paclitaxel-administered group (3b and 3B), and moderate expression in paclitaxel-administered groups treated with rutin (3c and 3C) and hesperidin (3d and 3D) and mild expression in the paclitaxel-administered group treated the combination of rutin and hesperidin (3e and 3E). Arrows indicate positive reactivity. 3f indicates the image analysis result of TNF-*α* of the tested groups. ^*a*^*p* < 0.05: significant compared with the normal group. ^*b*^*p* < 0.05: significant compared with the paclitaxel-injected group. ^*c*^*p* < 0.05: significant compared with the paclitaxel-injected group treated with both rutin and hesperidin. Photomicrographs 3A–3E are magnified sectors of Photomicrographs 3a–3e respectively.

As demonstrated in Figures 2 and 3, immunohistochemical detection of expressed caspase-3 and TNF-*α* in the liver was performed. Caspases-3 and TNF-*α* immunohistochemistry reactivity was very feeble in the liver sections of normal control rats, indicating that their expression levels are very low. Caspase-3 and TNF-*α* staining in the livers of paclitaxel-administered rats was highly positive, as shown by a dense cytoplasmic brownish-yellow color that suggested their high expression, with percentage changes of +549.29% and +309.55%, respectively, in comparison to the control group. Rutin, hesperidin, and their combination significantly reduced the enhanced caspase-3 activity and TNF-*α* concentration in paclitaxel-administered rats. The treatment of paclitaxel-administered rats with rutin and hesperidin combination was the most successful in lowering caspase-3 and TNF-*α* expressions.

## 4. Discussion

Paclitaxel is a drug that is commonly used to treat a variety of cancers. Its use may have a variety of adverse effects on several organs, including the liver, kidneys, and heart [67–70]. Despite remarkable progress in cancer research, compounds derived from natural resources are powerful candidates for cancer treatment [71]. Flavonoids and other reported phenolic components were discovered to have impressive antioxidative, cardioprotective, anticancer, antibacterial, antidiabetic, hypertensive, anti-inflammatory, and immune response enhancing effects as well as to protect skin from harmful ultraviolet radiation, making them outstanding drugs for pharmaceutical and medical use [72–74].

This study showed that the intraperitoneal injection of paclitaxel in the form of Taxol at a dose of 2 mg/Kg *b*. wt twice a week for 6 weeks caused hepatotoxicity, which was manifested biochemically by a significant increase in serum activities of cytosolic enzymes (ALT, AST, and LDH) due to their leakage into the bloodstream from injured hepatocytes [75]. Elevated serum ALT and AST levels in hepatocellular damage have been previously reported in paclitaxel-induced hepatotoxicity models [76–81]. Furthermore, the activity of LDH increased in paclitaxel-administered rats [82]. The LDH activity is elevated in patients with cancer and as a result of tissue damage; it is a common marker of toxicity. Additionally, we found a significant elevation in serum activities of membrane-bound enzymes (ALP and GGT) as a result of the increased rate of bile duct production and/or regurgitation in the blood after bile duct blockage [83]. These findings are similar to those reported by Ortega-Alonso et al. [84] who stated that the alteration of membrane permeability of liver cells and bile ducts triggers the release of their specific enzymes, notably GGT and ALP. Moreover, paclitaxel administration led to a significant increase in the total bilirubin content [85, 86], and this increase may be indicative of a specific liver injury and loss of function [87]. The serum albumin level was significantly reduced in paclitaxel-administered rats, which agrees with Wang et al. [88], who found that serum albumin concentration decreased significantly following chemotherapy. A decrease in albumin concentration, as observed in paclitaxel-administered rats, indicated insufficiency of albumin synthesis by the liver due to hepatopathy [89]. These biochemical parameter alterations strongly correlate with hepatic histopathological changes in the form of central vein and portal area blood vessel congestion, marked degenerative changes, including fatty changes and moderate necrotic changes with focal nuclear pyknosis in certain areas, focal leucocytic infiltration, and Kupffer cell proliferation. The current findings are congruent with those of Salahshoor et al. [80] who showed obvious changes and damage in the liver following paclitaxel treatment. Additionally, hepatotoxic effects following paclitaxel therapy were observed [90, 91]. It has been also found a distinctive hepatocellular carcinoma in hepatic histological sections in all groups following paclitaxel treatment was observed [85].

Rutin and/or hesperidin treatment of paclitaxel-administered rats successfully reduced increased blood ALT, AST, LDH, ALP, and GGT activities, as well as serum total bilirubin levels, by stopping further paclitaxel-induced hepatocellular damage and stabilizing membrane activity, thereby decreasing the leakage of these enzymes into the general circulation. The treatments potentially increased the reduced serum albumin level. Moreover, most hepatic histopathological changes were effectively improved by these treatments. Similar observations have been reported by Hozayen et al. [92] who stated that the pretreatment with rutin, hesperidin, and their combination can protect the liver against the hepatotoxic effect of doxorubicin by ameliorating the elevated AST, ALT, ALP, and *γ*-GT activities. This is attributed to the hepatoprotective potential of rutin [33] and hesperidin [34]. It was found that hesperidin reduces the severity of sodium arsenate (SA)-induced liver damage [93]. Rutin administration restored the elevated ALT, LDH, AST, and ALP levels in 5-fluorouracil (FU)-treated rats and improved the hepatic structure to normal [24]. Furthermore, rutin treatment improved carfilzomib-induced elevated levels of direct bilirubin in rats [94].

**Figure 4:**
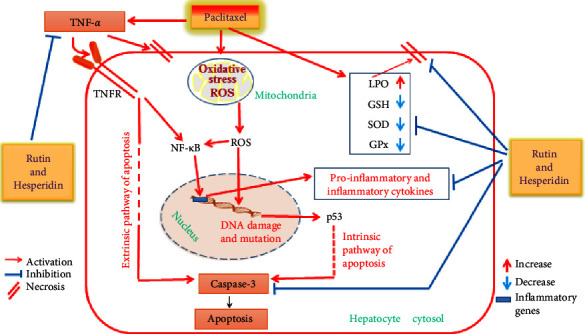
The effects of rutin and hesperidin on oxidative stress, inflammation, and apoptosis in the livers of paclitaxel-administered rats are depicted in a schematic diagram. The target effects of rutin and hesperidin on various mediators of oxidative stress, inflammation, and apoptosis are shown. The figure was designed by us using power point software.

Enzymatic and nonenzymatic antioxidant substances are components of antioxidant defense systems. GSH has a tripeptide structure and is a potent nonenzymatic antioxidant. SOD, catalase, and GPx are additional enzymatic antioxidants for ROS defense [95, 96]. Paclitaxel administration increases the formation of oxygen-free radicals, decreases antioxidants (SOD and GPx) and GSH content, and increases LPO, which results in liver damage. These results are consistent with those of Harisa [97] who reported that paclitaxel induces oxidative stress through decreased GSH content and increased MDA levels. In addition, it was reported that paclitaxel increases ROS and MDA concentrations and decreases SOD activity [82], indicating that paclitaxel induces changes in protein expression associated with apoptosis and ROS generation (Figure 4). ROS activates several mechanisms by damaging cell membranes and macromolecules in cells, resulting in inflammation and cell death [98]. Therefore, oxidative stress, which is caused by paclitaxel administration, may cause the production of active oxygen species, including pure oxygen, H_2_O_2_ and superoxide radicals, which destroy cells, DNA, proteins, and intracellular lipids, and finally liver damage [99]. According to the findings, rutin and hesperidin treatment remarkably reduced paclitaxel-induced oxidative stress by reducing LPO and improving GSH content along with the activities of antioxidant enzymes due to the ability of rutin to recover-free radicals by chelating metallic iron ions [100, 101] as well as the antioxidant activity and radical recovery properties of hesperidin [102, 103]. These findings are consistent with those of Hozayen et al. [92], who found that rutin and hesperidin significantly increased GSH and GPx levels in the liver and decreased the LPO level in doxorubicin-treated rats. Rutin treatment alleviated liver and kidney damage by reducing oxidative stress, endoplasmic reticulum stress, inflammation, apoptosis, and autophagy caused by valproic acid [104]. Additionally, rutin has a hepatoprotective role in eliminating isoniazid-induced oxidative stress [33]. Hesperidin has been discovered to protect the brain, liver, kidneys, and oxidative damage caused by numerous toxins [105, 106]. In another way, thymoquinone and costunolide are also natural products that have been shown to have an apoptotic effect to rapidly eliminate the senescent cells induced by doxorubicin and induce apoptosis of proliferative cancer cell lines [107].

Immunohistochemical investigations showed a significant increase in the proapoptotic protein (caspase-3) activity and pro inflammatory cytokine (TNF-*α*) concentration in the liver of paclitaxel-administered rats. The findings of our investigation agree with those of Yardım et al. [108] who revealed that the mRNA levels of TNF-*α* and caspase-3 were higher in the paclitaxel group for the sciatic nerve and spinal cord, and the immunohistochemical expression of caspase-3 in the paclitaxel-induced bone marrow tissue was increased. Furthermore, taxanes, including paclitaxel, induced an increase in IL-1*β*, IL-6, and TNF-*α* levels in patients with cancer [109–111]. It was also found that circulating IL-6 and TNF-*α* levels were increased 3 days after a 6-dose paclitaxel regimen [112]. TNF-*α* is a critical mediator of inflammation [113] that has been demonstrated to recruit and trigger more inflammatory cells in response to increased oxidative stress [114]. TNF-*α* can promote hepatocyte apoptosis *via* binding to TNF receptors (TNFR) and death receptors, triggering the extrinsic apoptosis pathway [115–117] (Figure 4). Through the permeability of the mitochondrial membrane or its transition pore apertures, paclitaxel releases apoptogenic components, including cytochrome C, into the cytosol, either directly or indirectly [118, 119]. Apoptosis is facilitated by cytochrome C active caspase-9, which stimulates various caspase enzymes, including caspase-3 and caspase-7, in the presence of apoptotic protease activating factor-1 [120, 121].

The treatment of paclitaxel-administered rats with rutin and/or hesperidin suppressed the activity of caspase-3, which is a common mediator of extrinsic and intrinsic apoptotic pathways and the level of TNF-*α*, which is a key regulator of inflammation (Figure 4). These results are consistent with those of Li and Schluesener [122] who reported that hesperidin suppressed oxidative/nitrative stress, inflammation, and apoptosis. Hesperidin reduced the caspase-3 activity and showed an anti-inflammatory effect by decreasing the levels of TNF-*α*, nuclear factor kappa B (NF-*κ*B), and IL1*β* in the kidney and liver tissues of rats with SA-induced toxicity [93]. It also reduced the serum level of TNF-*α* in arthritic rats [123]. Hesperidin decreased the elevated liver caspase-3 expression and altered serum TNF-*α*, IL-17, and IL-4 levels in diclofenac-administered rats [124]. Additionally, rutin may have potential protective benefits against hepatotoxicity induced by doxorubicin through reducing oxidative stress, inflammation, and apoptosis as well as altering the expression of the nuclear factor erythroid 2–related factor 2 (Nrf2) gene [125]. Rutin decreased the hepatic TNF-*α* and IL-6 levels of carbon tetrachloride-treated rats [126]. It was found that rutin significantly decreased caspase-3 immunopositivity in 5-FU-treated rats [24]. The therapeutic potential of rutin can be owed to its antioxidant, anti-inflammatory, antiallergic, and antiangiogenic properties [127, 128]. Based on our findings and past research studies, the intrinsic pathway, which is activated by high ROS levels, or extrinsic ligands of pathway receptors, such as TNF-*α*, can cause caspase-3, the apoptosis executor, to be activated in paclitaxel hepatotoxicity. Rutin and hesperidin may have reduced apoptosis by modulating both intrinsic and extrinsic apoptotic pathways by suppressing oxidative stress and significantly lowering increased TNF-*α* concentration (Figure 4). In addition, TNF-*α* (through canonical pathway) can activate NF-*κ*B, which promotes NF-*κ*B target genes involved in inflammatory responses [129]. Both rutin and hesperidin may produce their anti-inflammatory effects by affecting the canonical pathway of NF-*κ*B through the suppression of TNF-*α* levels and in turn inhibition of TNF-*α* receptors (TNFR) (Figure 4).

## 5. Conclusion

Oral administration of rutin, hesperidin, and their combination could counteract paclitaxel-induced liver damage and toxicity by strengthening the antioxidant defense system and decreasing oxidative stress and apoptosis. Additionally, it was discovered that rutin and hesperidin combined therapy was the most effective at restoring liver function and histological integrity in paclitaxel-administered rat models. However, before rutin and hesperidin be used in humans, more clinical trials are necessary to evaluate their effectiveness and safety during paclitaxel administration. The Food and Drug Administration also needs to approve their use in human beings these evaluations. Moreover, further studies are required to scrutinize the effect on mediators of apoptosis other than caspase-3 and mediators of inflammation other TNF-*α* to identify other targets of rutin and hesperidin in paclitaxel-administered rats.

## Figures and Tables

**Table 1:** Effects of rutin and hesperidin on the activities of serum enzymes related to liver function in paclitaxel-injected rats.

Groups	*Parameters*													
	AST (U/L)	% Change	ALT (U/L)	% Change	GGT (U/L)	% Change	LDH (U/L)	% Change	ALP(U/L)	% Change	Total bilirubin (mg/dl)	% Change	Albumin (g/dl)	% Change
Normal	120.67 ± 4.56	—	41.17 ± 2.94	—	5.10 ± 0.45	—	565.50 ± 29.48	—	183.50 ± 6.89	—	0.23 ± 0.03	−	2.97 ± 0.16	—
Paclitaxel	185.00 ± 11.17^a^	53.31	89.83 ± 2.68^a^	118.19	10.80 ± 0.60^a^	111.76	2327.50 ± 122.24^a^	311.58	781.00 ± 34.58^a^	325.61	0.76 ± 0.12^a^	230.43	1.86 ± 0.10^a^	−37.37
Paclitaxel + rutin	132.50 ± 6.01^b^	−28.38	51.60 ± 2.23^abc^	−42.56	7.80 ± 0.48^ab^	-27.78	1681.67 ± 115.22^ab^	−27.75	307.33 ± 21.87^ab^	−60.65	0.33 ± 0.03^bc^	−57.69	2.45 ± 0.14^b^	31.72
Paclitaxel + hesperidin	145.13 ± 5.14^b^	−21.55	61.80 ± 1.89^abc^	−31.20	7.20 ± 0.48^ab^	-33.33	1515.83 ± 167.47^ab^	−34.87	368.67 ± 39.30^ab^	−52.79	0.30 ± 0.02^bc^	−61.54	2.43 ± 0.11	30.65
Paclitaxel + rutin + hesperidin	131.50 ± 9.10^b^	−28.92	43.92 ± 2.22^b^	−51.11	7.50 ± 0.18^ab^	-30.56	988.33 ± 187.86^b^	−57.54	284.67 ± 25.89^b^	−63.55	0.25 ± 0.01^b^	−67.95	2.50 ± 0.17^b^	34.41

Data are expressed as Mean ± SEM (*n* = 6). ^*a*^*p* < 0.05: significant compared with the normal group. ^*b*^*p* < 0.05: significant compared with the paclitaxel-injected group. ^*c*^*p* < 0.05: significant compared with the paclitaxel-injected group treated with both rutin and hesperidin. Percentage changes are calculated by comparing the paclitaxel-injected group with normal and paclitaxel-injected groups treated with rutin and hesperidin with the paclitaxel-injected group.

**Table 2:** Effects of rutin and hesperidin on liver LPO, GSH content, and activities of SOD and GPx in paclitaxel-injected rats.

Groups	*Parameters*							
	LPO (nmol MDA/100 mg tissue/hour)	% Change	GSH (nmol/100 mg tissue)	% Change	SOD (U/g tissue)	% Change	GPx (mU/100 mg tissue)	% Change
Normal	11.10 ± 0.62	—	87.68 ± 3.32	—	19.05 ± 0.18	—	99.70 ± 1.80	—
Paclitaxel	23.30 ± 2.05^a^	109.91	52.89 ± 1.96^a^	−39.67	17.18 ± 0.13^a^	−9.80	87.20 ± 1.20^a^	−12.54
Paclitaxel + rutin	18.10 ± 0.64^ab^	−22.32	75.83 ± 2.24^abc^	43.37	18.16 ± 0.14^ab^	5.70	93.90 ± 1.10^ab^	7.68
Paclitaxel + hesperidin	12.10 ± 1.30^bc^	−48.10	71.86 ± 0.85^abc^	35.86	18.37 ± 0.09^ab^	6.93	92.70 ± 0.80^ab^	6.31
Paclitaxel + rutin + hesperidin	18.10 ± 0.96^ab^	−22.32	54.17 ± 2.89^a^	2.42	18.46 ± 0.12^ab^	7.46	92.20 ± 0.60^ab^	5.73

Data are expressed as Mean ± SEM (*n* = 6). ^*a*^*p* < 0.05: significant compared with the normal group. ^*b*^*p* < 0.05: significant compared with the paclitaxel-injected group. ^*c*^*p* < 0.05: significant compared with the paclitaxel-injected group treated with both rutin and hesperidin. Percentage changes are calculated by comparing the paclitaxel-injected group with normal and paclitaxel-injected groups treated with rutin and hesperidin with the paclitaxel-injected group.

**Table 3:** Pathological hepatic lesion scores in different groups.

Groups	*Parameters*					
	Degenerative change	Fatty change	Necrosis	Inflammatory cells	Congestion	Activated Kupffer cell proliferation
Normal	0	0	0	0	0	0
Paclitaxel	3.83 ± 0.17^a^	3.83 ± 0.17^a^	2.17 ± 0.17^a^	3.33 ± 0.21^a^	3.17 ± 0.4^a^	3.67 ± 0.21^a^
Paclitaxel + rutin	2.50 ± 0.22^abc^	3.00 ± 0.26^abc^	1.00 ± 0.37^ab^	1.83 ± 0.31^ab^	1.50 ± 0.22^ab^	2.67 ± 0.33^ab^
Paclitaxel + hesperidin	3.00 ± 0.37^abc^	2.83 ± 0.31^abc^	1.67 ± 0.31^ab^	2.00 ± 0.36^ab^	1.67 ± 0.33^ab^	2.50 ± 0.22^ab^
Paclitaxel + rutin + hesperidin	1.67 ± 0.33^ab^	2.00 ± 0.26^ab^	1.33 ± 0.21^ab^	1.50 ± 0.43^ab^	1.00 ± 0.26^ab^	2.17 ± 0.31^ab^

Data are expressed as Mean ± SEM (*n* = 6). ^*a*^*p* < 0.05: significant compared with the normal group. ^*b*^*p* < 0.05: significant compared with the paclitaxel-injected group. ^*c*^*p* < 0.05: significant compared with the paclitaxel-injected group treated with both rutin and hesperidin. Scoring of hepatic histological lesions was calculated and graded as follows 0 = none; 1 ≤ 25%; 2 = 26–50%; 3 = 51–75%; and 4 = 76–100%.

## Data Availability

All data are available from the corresponding author upon reasonable request.

## Abbreviations

ALP: Alkaline phosphatase
ALT: Alanine aminotransferase
ANOVA: One-way analysis of variance
AST: Aspartate aminotransferase
CMC: Carboxymethylcellulose
DAB: 3,3′-Diaminobenzidine
DNA: Deoxyribonucleic acid
GGT: Gamma-glutamyl transferase
GPx: Glutathione peroxidase
GSH: Reduced glutathione
GSSG: Oxidized glutathione
H&E: Hematoxylin and eosin stain
H_2_O_2_: Hydrogen peroxide
IFN-*γ*: Interferon-*γ*
IL: Interleukin
b: Wt: Kilogram body weight
LDH: Lactate dehydrogenase
LPO: Lipid peroxidation
MDA: Malondialdehyde or 1,1,3,3-tetramethoxypropane
mRNA: Messenger ribonucleic acid
NF-*κ*B: Nuclear factor kappa B
Nrf2: The nuclear factor erythroid 2–related factor 2
O^2−^: Superoxide radical
rpm.: Round per minute
ROS: Reactive oxygen species
SA: Sodium arsenate
SEM: Standard error of the mean
SPSS: Statistical Package for Social Sciences
SOD: Superoxide dismutase
TBA: Thiobarbituric acid
TCA: Trichloroacetic acid
TNFR: TNF receptor
TNF-*α*: Tumor necrosis factor-alpha.
